# Technical Consideration towards Robust 3D Reconstruction with Multi-View Active Stereo Sensors

**DOI:** 10.3390/s22114142

**Published:** 2022-05-30

**Authors:** Mingyu Jang, Seongmin Lee, Jiwoo Kang, Sanghoon Lee

**Affiliations:** 1 Department of Electrical and Electronic Engineering, Yonsei University, Seoul 03722, Korea; jmg1002@yonsei.ac.kr (M.J.); lseong721@yonsei.ac.kr (S.L.); 2 Department of IT Engineering, Sookmyung Women’s University, Seoul 04310, Korea; 3 Department of Radiology, College of Medicine, Yonsei University, Seoul 03722, Korea

**Keywords:** multi-view active stereo sensors, RGB-D sensor, 3D reconstruction, multi-sensor scanning system construction

## Abstract

It is possible to construct cost-efficient three-dimensional (3D) or four-dimensional (4D) scanning systems using multiple affordable off-the-shelf RGB-D sensors to produce high-quality reconstructions of 3D objects. However, the quality of these systems’ reconstructions is sensitive to a number of factors in reconstruction pipelines, such as multi-view calibration, depth estimation, 3D reconstruction, and color mapping accuracy, because the successive pipelines to reconstruct 3D meshes from multiple active stereo sensors are strongly correlated with each other. This paper categorizes the pipelines into sub-procedures and analyze various factors that can significantly affect reconstruction quality. Thus, this paper provides analytical and practical guidelines for high-quality 3D reconstructions with off-the-shelf sensors. For each sub-procedure, this paper shows comparisons and evaluations of several methods using data captured by 18 RGB-D sensors and provide analyses and discussions towards robust 3D reconstruction. Through various experiments, it has been demonstrated that significantly more accurate 3D scans can be obtained with the considerations along the pipelines. We believe our analyses, benchmarks, and guidelines will help anyone build their own studio and their further research for 3D reconstruction.

## 1. Introduction

The demand for accurate reconstruction of three-dimensional (3D) objects has been increasing recently in various fields [1,2,3,4,5,6,7], such as computer vision, computer graphics, robotics, and image processing. However, 3D and four-dimensional (4D) scanning devices that accurately reconstruct 3D objects are still prohibitively expensive for widespread use. Fortunately, with affordable off-the-shelf sensors, RGB-D sensors, such as Microsoft Kinect [8] and Intel RealSense [9], are widely available, allowing users to affordably construct 3D and 4D scanning systems using multiple sensors. However, the quality of these system products [10,11,12,13,14,15] is sensitive to numerous factors, such as how well-calibrated the system is, depth estimation, 3D reconstruction, and color mapping accuracy. This paper presents technical investigations along 3D reconstruction pipelines with active stereo sensors. The quality of reconstructed 3D shapes is closely correlated with the details of each pipeline, as the pipelines consecutively contribute to reconstructing 3D shapes. This study helps users construct accurate and robust 3D reconstruction systems with multiple RGB-D sensors, especially active stereo sensors, by providing various technical considerations through the pipelines.

**Figure 1:**
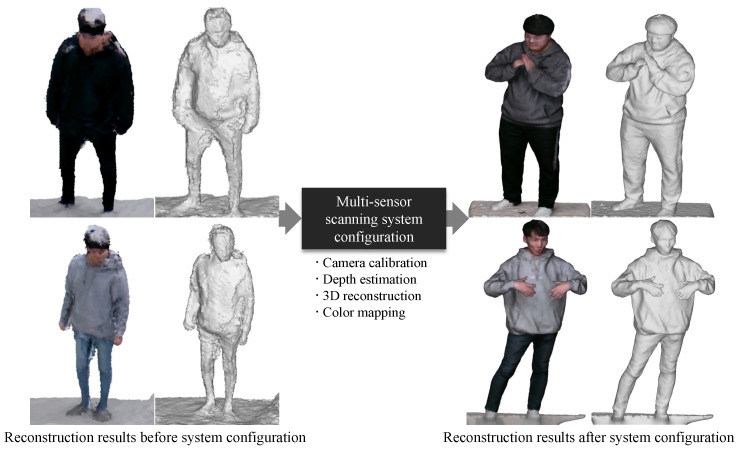
The significance of the proper configuration for multi-sensor scanning systems to accomplish a high-quality 3D reconstruction.

Active stereo scanning systems are sensitive to various factors, so they need to be approximately configured. Figure 1 shows quality comparisons between 3D reconstructions captured from multi-sensor systems before and after the adequate configuration. Systems with the same hardware specifications can produce products with significantly different levels of quality in geometric and color details. Thus, it is necessary to properly configure active stereo scanning systems considering various technical factors to achieve high-quality reconstruction results.

We have already examined and discussed the performances of various stereo matching algorithms on active stereo pairs [16]. Nevertheless, they were analyses on single stereo pairs, not correlating with other stereo pairs. Several considerations are required to obtain high-quality 3D meshes because there are consecutive pipelines correlated with each other to reconstruct 3D meshes from multiple active stereo sensors. This paper presents difficulties and considerations in constructing a reconstruction system using multi-sensors and provides practical guidelines to reconstruct accurate and reliable 3D surfaces. Moreover, the variable factors that can significantly affect the reconstruction quality were carefully examined in the reconstruction procedures that use multiple active stereo sensors. The overall pipeline of 3D reconstruction using multiple RGB-D sensors is composed of several sub-procedures: *RGB-D camera calibration, projector intensity, stereo matching algorithm, 3D reconstruction, outlier removal, and color mapping*. In each procedure, technical considerations were analyzed and benchmarks were performed using data captured by 18 recent RGB-D sensors. Based on these technical considerations, this paper provides the guidelines to obtain high-quality 3D surfaces from the reconstruction system; it has been demonstrated that significantly more accurate 3D scans can be obtained with proper consideration. To the best of our knowledge, this is the first (and detailed) set of guidelines that presents the entire pipeline (along with performance comparisons) for robust 3D reconstruction with multi-view active stereo sensors. The overview, benchmarks, and solutions of the reconstruction procedures can help people to build their own reconstruction studios using multiple active stereo sensors. In summary, the key contributions of this paper are summarized as follows:

- This paper presents the entire 3D reconstruction pipeline from multi-view active stereo sensors. To the best of our knowledge, this is the first and most detailed set of guidelines for 3D reconstruction with multi-view active stereo sensors.
- The reconstruction pipeline was divided into sub-procedures; various technical factors that could significantly affect the reconstruction accuracy were thoroughly examined in each sub-procedure.
- Through the experiments, this paper provides practical guidelines to reconstruct accurate and reliable 3D objects.

## 2. Related Work

Commercial RGB-D sensors commonly use either time-of-flight (ToF) [17] or active stereo techniques [18,19] to estimate object depth. ToF sensors consist of emitters and receivers. They measure object depths according to the amount of time that passes from when a signal is emitted by the sensor to when it is received. In contrast, the active stereo sensors, consisting of stereo image sensors and a projector, calculate the depth by finding point correspondences between captured stereo images; the additional texture is supplied to the object’s surfaces for reliable matching by emitting unknown light patterns to the object using a projector.

The active stereo technique is more appropriate for multi-sensor scanning systems than the ToF technique for three significant reasons. The first reason is that the number of sensors in the system is positively correlated with reconstruction accuracy in active stereo systems but negatively in ToF systems. In active stereo systems, each sensor projects its pattern on the object, creating more complex textures and allowing the system to more easily identify corresponding points in the images [20,21,22]. The opposite effect occurs in ToF systems because ToF sensors interfere with each other. The second reason is that commercial active stereo sensors can be adjusted in more ways than ToF sensors because ToF system characteristics are largely hardware-dependent. Many parameters, such as projector intensity, sensor gain, and matching algorithm, can be changed in active stereo sensors and be used in a wide variety of situations [23]. They offer significant benefits (e.g., multi-sensor scanning systems to meet user demands). The third reason is that active stereo sensors have higher resolutions than ToF sensors because active stereo sensors use high-resolution cameras [9]. For example, Intel RealSense D455 (30 FPS, 1280 × 720) [20], which is the most popular active stereo sensor, has a higher resolution than Microsoft Azure Kinect (30 FPS, 640 × 576) [8], which is the most widely used ToF sensor, even if they are similar in price. Capturing a high-resolution depth map is important to reconstruct accurate 3D objects.

For these reasons, multi-sensor scanning systems should use active stereo sensors rather than ToF sensors.

## 3. 3D Reconstruction Framework with Multi-View Active Stereo Sensors

**Figure 2:**
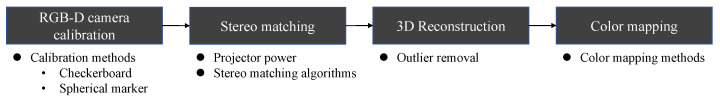
Overall pipeline of 3D reconstruction and technical factors that affect the reconstruction quality in each sub-procedure.

To accurately reconstruct a 3D object from multiple RGB-D sensors, this paper examines and benchmarks multi-view reconstruction procedures with various factors that can significantly affect the reconstruction quality. Figure 2 shows the 3D reconstruction procedures and the variable factors that could affect the reconstruction quality. The procedures are discussed in the following order: (A) *RGB-D camera calibration*, (B) *projector intensity*, (C) *stereo matching algorithm*, (D) *3D reconstruction*, (E) *outlier removal*, and (F) *color mapping*. Before capturing objects, multiple sensors must be synchronized using an external trigger to capture RGB-D images from the sensors simultaneously [24]. The *camera calibration* techniques estimate the camera’s intrinsic and extrinsic parameters for generating 3D points in the camera’s local coordinates (from depth maps) and transform them into points in the global coordinates. After capturing objects, the depth is estimated by finding the image corresponding from a pair of infrared (IR) images captured from IR sensors via the *stereo matching algorithm*. The 3D points estimated in the local sensor coordinates of each calibrated sensor are transformed into the global coordinates and combined with 3D points from the other sensors. The *3D reconstruction* is the process of generating a 3D object’s surface from the incorporated point clouds. However, if the 3D points predicted from noisy depths are incorporated without pre-processing, significant artifacts can arise in the point cloud and the reconstructed surface. These noisy points can be effectively removed by using multi-view consistency. Finally, the colors of the 3D reconstructed mesh are obtained by reprojecting the 3D vertices of the mesh to the RGB images. In the following sections, the details, challenges, and solutions of each procedure are presented.

## 4. Technical Considerations toward Robust 3D Reconstruction

This paper examines which variable factors in the sub-procedures affect the 3D reconstruction quality to provide accurate guidance for constructing a 3D reconstruction system. All evaluations and experiments to analyze the influence of technical variable factors were performed using 18 *RealSense D455* (N=18), which is the recent RGB-D sensor using an active stereo (Intel, Dallas, TX, USA). The RGB and depth streams were captured in HD resolutions. In detail, the RGB stream was captured at a resolution of 1280×800; a depth stream was captured at a resolution of 1280×720. The RGB-D sensors were configured to cover 360 degrees of a target object with 60-degree intervals and three different heights. The sensor installed heights were 30, 90, and 150 cm from the ground, respectively.

Six desktop computers with *Intel Core i7 CPUs* and single *Nvidia Geforce GTX 2080 Ti* GPUs were used to capture the sensors by connecting three sensors to one PC, to cover the high bandwidth requirements from the sensors. This paper used *KOTRON TG-16C* and *KOTRON TG-4C* (KOTRON, Seoul, Korea) external synchronization devices to capture the sensors simultaneously. The software was developed in *C++* with *OpenCV* [25] and *Intel RealSense SDK 2.0* [23].

### 4.1. RGB-D Camera Calibration

**Figure 3:**
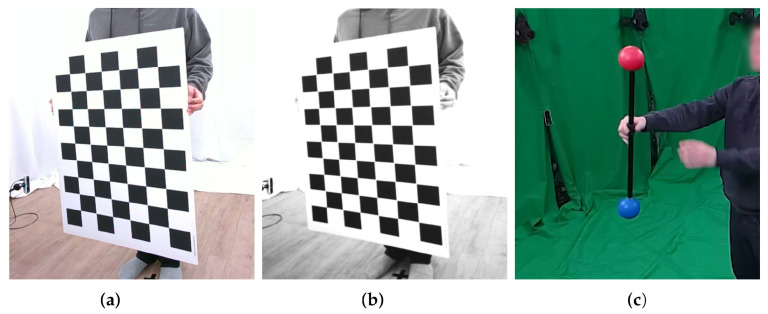
Two kinds of calibration objects: (**a**) checkerboard, (**b**) checkboard of IR domain and (**c**) spherical marker. The calibration object is clearly visible in an IR sensor (**b**) by increasing the sensor gain sufficiently.

Each sensor, consisting of a pair of IR sensors and a single RGB sensor, produced images of two IR and one RGB by capturing objects in the multi-view capture environment. The RGB and IR sensors were calibrated using image point correspondences among them. The correspondence points can be made by simultaneously capturing a calibration object in Figure 3 and detecting features or corner points in the captured images. Increasing the IR sensor’s gain and using external IR light sources help the IR sensor to capture a calibration object clearly, as described in Figure 3b.

According to the types of calibration objects used in the camera calibration, as described in Figure 3, the calibration methods can be classified into two categories: the checkerboard-based method [26] and spherical marker-based method [27,28]. Checkerboards have been widely used for camera calibration because their corners can be easily and clearly detected using prior information. The checkerboard-based method estimates the 3D positions of the checkerboard corners by inferring the 3D structure from the 2D images using the known number of squares and the side lengths of squares in the checkerboard as prior information. However, most cameras in a multi-view setup cannot capture the checkerboard simultaneously because the planar checkerboard is only visible in the frontal view. In contrast, the spherical marker-based method [27] uses spherical [27] or optical [28] markers to simultaneously capture the marker points from multiple cameras.

Here, the accuracy of one standard checkerboard-based calibration method [26] and two widely used spherical marker-based methods [27,28] for the multi-view camera calibration are compared. This paper used the OpenCV implementation [25] for the checkerboard method and official implementations from the authors for spherical marker-based ones.

Table 1 shows the reprojection errors [29] of multiple sensors by means of the root mean square error (RMSE) for three calibration methods. The method by Mitchelson et al. [28] uses a calibration object composed of two spherical markers with fixed distances. In contrast, the method by Svoboda et al. [27] requires one point per image. The prior distance between the markers of Mitchelson’s method helps obtain a lower error than one obtained from Svoboda’s method. The checkerboard outperforms the spherical marker-based methods, demonstrating that strong checkerboard priors are significantly beneficial for obtaining accurate and reliable calibration results.

### 4.2. Projector Intensity

This paper examines how the projector intensity of the pattern projected on the object’s surface as part of depth scanning affects the quality of the 3D reconstruction. For example, in the captured IR image, we can clearly observe both pattern dots from the projector and the textures of the target object under an approximate projector intensity. However, the high projector intensity makes the pattern dots overwhelm the textures of the target. In contrast, the low projector intensity apparently makes it difficult to distinguish between the pattern dots and the textures of the target. Since depth maps are calculated by matching IR image correspondences, the intensities and contrasts of these pattern dots and object textures on the object surface play major roles in the matching resolution. It can be assumed that the depth quality is highly dependent on the projector intensity. Thus, this paper evaluates quantitative and qualitative results by changing the projector intensity from 30 to 360 in increments of 30, which is served from *Intel RealSense SDK 2.0*. Recent commercial RGB-D sensors using active stereo techniques [9], *Intel RealSense D455*, were used for the evaluation.

**Figure 4:**
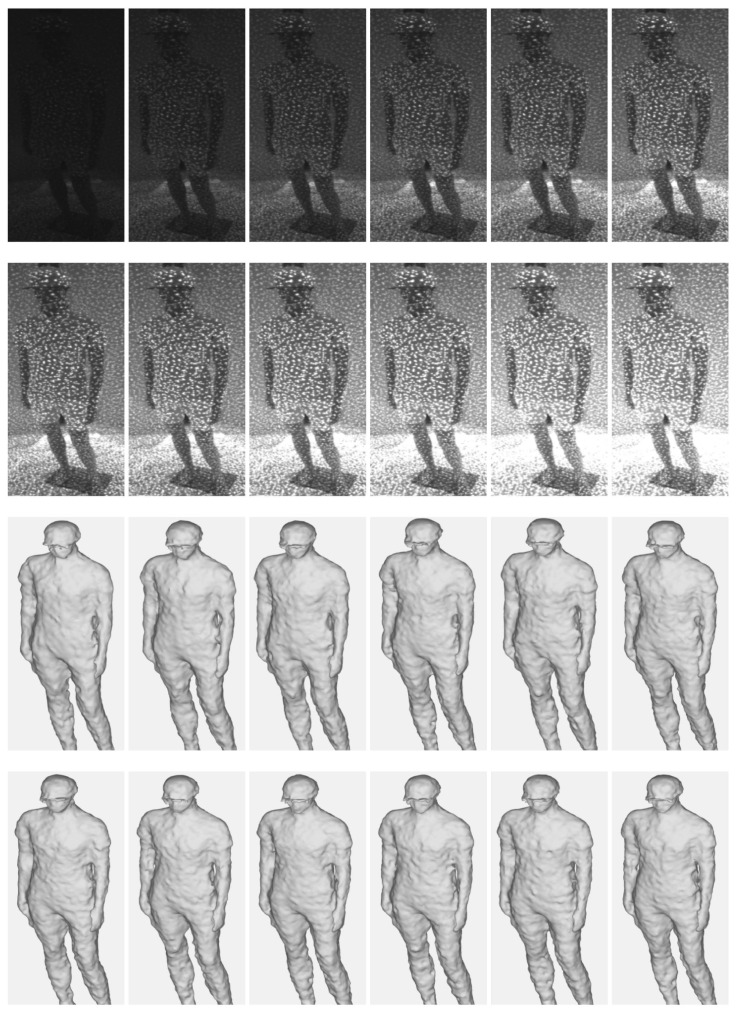
Captured IR images and 3D reconstructed results using multi-sensors according to projector intensity. The intensity ranges from 30 to 360 and increases by an interval of 30, from left to right.

Figure 4 shows 3D reconstruction results from multi-view depths according to the IR projector intensity; Table 2 summarizes the reprojection errors to the depths by means of RMSE. Note that the census transform-based stereo matching scheme [30] was used to estimate the depth in the RealSense depth sensor. A meaningful difference between the results in Figure 4 and Table 2 was not found in our tests. The results captured using various projector intensities do not show significant differences, except when the IR projector’s intensity is too strong or weak.

The comparison results demonstrate that the texture of an object itself does not significantly affect the quality of the stereo matching in an active stereo system. Thus, the results imply that the projector pattern dots have the dominant information rather than the object’s textures for matching the corresponding points between IR stereo images.

### 4.3. Stereo Matching Algorithm

It is reasonable to consider that the stereo matching algorithm used to match IR stereo images plays a significant role in the quality of depth because the depth is calculated from disparity [31], which is the coordinate differences of the points. Therefore, this paper examines the depth estimation qualities according to the stereo matching algorithms. Mainly, stereo matching algorithms are categorized into two types: patch-based methods [30,32,33] and deep learning-based methods [34,35].

**Figure 5:**
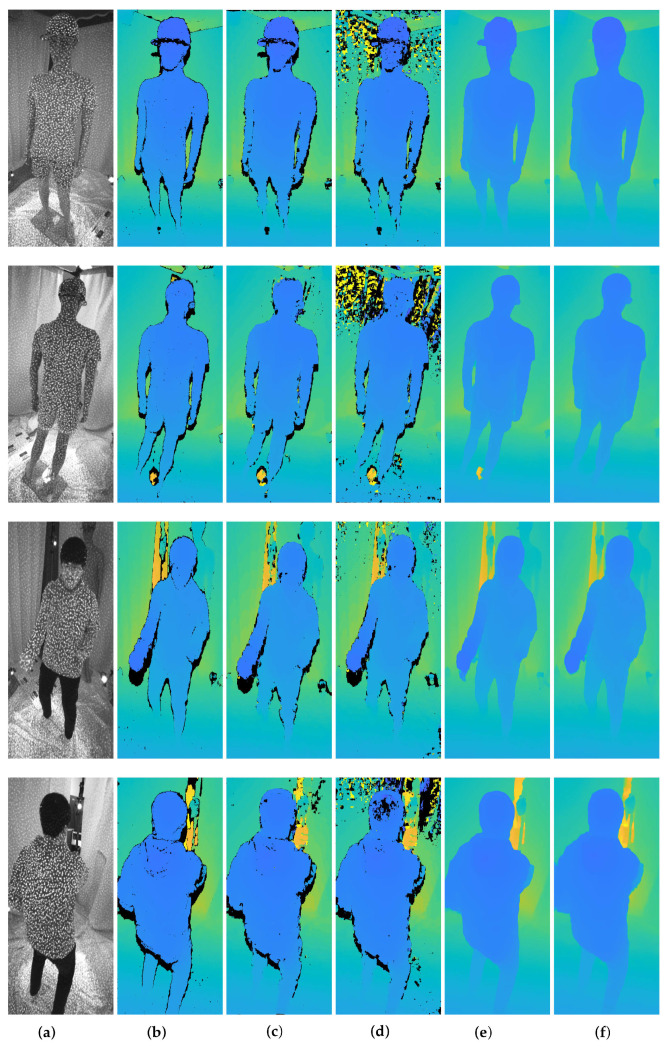
Comparisons of the depth estimations among stereo matching algorithms. (**a**) Infra (Left); (**b**) Census; (**c**) NCC; (**d**) SSD; (**e**) AANet; (**f**) DeepPruner.

Figure 5 depicts depth maps estimated by five stereo matching methods: census transform (Census) [30], normalized cross correlation (NCC) [32], sum of squared differences (SSD) [33], adaptive aggregation networks (AANet) [34], and DeepPruner [35]. In the results, Census and AANet methods show notably better results over the other methods. Census uses a non-parametric transformation that does not depend on the actual value of the image intensity; it only depends on the relative differences in the intensities. It enables robust depth estimation in images with the variation of illumination caused by the IR projector. On the other hand, using deformable convolution layers [36,37] that dynamically determine offsets and a scale of a convolutional filter according to inputs, AANet accurately estimates depths from active stereo images by flexibly coping with pattern dots. In the experiments on the projector intensity in Section 4.2, it was found that the pattern dots from the IR projector worked as the dominant factors to determine the quality of depth. In a similar vein, Census and AANet stereo matching methods outperformed performances because they could distinguish between the pattern dots and their backgrounds, compared to other methods.

### 4.4. 3D Reconstruction

Three-dimensional (3D) points in the local sensor coordinates were reconstructed from a depth map using an intrinsic camera parameter. Subsequently, the 3D points in sensors were aligned into the global coordinates from the local sensor coordinates. Assuming *N* stereo sensors (1≤i≤N) and the depth map $D_{i}$ estimated in the ith stereo sensor, let $u_{i}^{j}={\left[u,v\right]}^{⊤}$ and $x_{i}^{j}={\left[x,y,z\right]}^{⊤}$ be a jth 2D pixel point of $M_{i}$ points ($1\leq j\leq M_{i}$) in the depth image plane and a jth 3D point in the local sensor coordinates, respectively. The 3D points in the local sensor coordinates were calculated from the depth map and intrinsic parameters as:

(1) $$\begin{array}{cc} x_{i}^{j} & =\lambda_{i}^{j}K_{i}^{-1}\left[u_{i}^{j},\;1\right], \end{array}$$

(2) $$\begin{array}{cc} \lambda_{i}^{j} & =D_{i}\left(u_{i}^{j}\right), \end{array}$$

where $K_{i}\in R^{3\times 3}$ and $\lambda_{i}^{j}$ are the intrinsic matrix and projective depth of the ith sensor, respectively. Moreover, a surface normal $n_{i}^{j}$ of the 3D point in the local sensor coordinates was computed using the cross product of the difference between the neighboring pixel values in the depth map as:

(3) $$\begin{array}{c} g_{x}=D_{i}\left(u+1,v\right)-D_{i}\left(u-1,v\right), \end{array}$$

(4) $$\begin{array}{c} g_{y}=D_{i}\left(u,v+1\right)-D_{i}\left(u,v-1\right), \end{array}$$

(5) $$\begin{array}{c} n_{i}^{j}=\frac{g_{x}\times g_{y}}{|g_{x}\left|\right|g_{y}|}, \end{array}$$

where $g_{x}$ and $g_{y}$ are depth gradients of the 2D pixel point $u_{i}^{j}$. To reconstruct the entire geometry from multiple depth maps estimated in multi-view cameras, the 3D points in the local sensor coordinates were integrated into the global coordinates. The 3D point $x_{i}^{j}$ and the surface normal $n_{i}^{j}$ of the ith sensor can be transformed into the 3D point $X_{i}^{j}\in R^{3}$ and the surface normal $N_{i}^{j}\in R^{3}$ of the global coordinates using the extrinsic matrix of the calibrated depth sensor, respectively, as:

(6) $$\begin{array}{cc} X_{i}^{j} & =R_{i}^{-1}\left(x_{i}^{j}-t_{i}\right), \end{array}$$

(7) $$\begin{array}{cc} N_{i}^{j} & =R_{i}^{-1}n_{i}^{j}, \end{array}$$

where $R_{i}\in R^{3\times 3}$ and $t_{i}\in R^{3\times 1}$ are the rotation matrix and translation vector of the ith sensor, respectively. Let a set of 3D points from the ith sensor be $X_{i}=\left[X_{i}^{1},\cdots ,X_{i}^{M_{i}}\right]\in R^{3\times M_{i}}$ and a set of normals in the global coordinates be $N_{i}=\left[N_{i}^{1},\cdots ,N_{i}^{M_{i}}\right]\in R^{3\times M_{i}}$. The entire vertices and normals from cameras are represented by $X=\left[X_{1},\cdots ,X_{N}\right]\in R^{3\times K}$ and $N=\left[N_{1},\cdots ,N_{N}\right]\in R^{3\times K}$, where $K=\sum_{i}M_{i}$. After that, the Poisson reconstruction algorithm [38] is used to reconstruct the surface of the object, using the vertices and normals, X and N.

**Figure 6:**
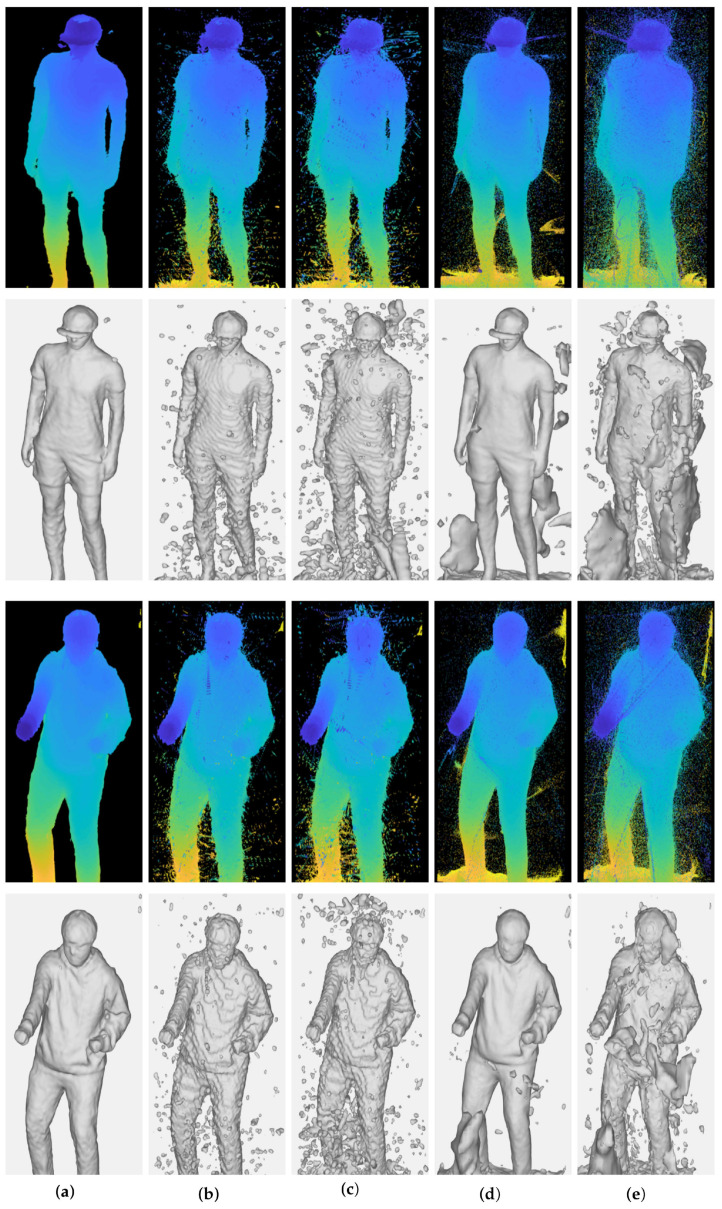
Qualitative results of 3D reconstruction according to the stereo matching algorithm. (**a**) Census; (**b**) NCC; (**c**) SSD; (**d**) AANet; (**e**) DeepPruner.

Figure 6 shows 3D point clouds and 3D meshes from multi-view depth maps generated by stereo matching algorithms in Section 4.3. Table 3 summarizes reprojection errors to the depths by means of RMSE. It is shown that high-quality depth maps in Figure 5 lead to better integration and reconstruction results. However, artifacts arise in several regions of results regardless of the methods used to estimate the depth maps.

The depths estimated by stereo matching algorithms can have erroneous values due to mismatching. In particular, deep learning-based methods produce uneven and inaccurate depths from image backgrounds. The accumulation of these erroneous points in the global coordinate leads to significant artifacts. Thus, the inaccurate points need to be excluded before the integration procedure to obtain accurate and precise reconstruction results.

### 4.5. Outlier Removal

Even a relatively small depth noise of a view can create a significant artifact in 3D space during the fusion of multi-view depth maps. Therefore, this paper introduces an outlier removal method for reliable and accurate reconstruction to efficiently handle the noisy depths using the multi-view consistency. This paper formulates the multi-view consistency based on distance and view consistencies. The framework determines a vertex to be valid if the distance between the projected vertex to each view and its depth value is smaller than the distance threshold $\epsilon_{d}$. Each vertex is determined as an inlier when the number of valid views is more than the view threshold $\epsilon_{v}$. Therefore, the vertex is determined to be valid under the following conditions:

(8) $$\begin{array}{c} \sum_{i=1}^{N}B\left(K_{i},R_{i},t_{i}\right)>\epsilon_{v}, \end{array}$$

(9) $$\begin{array}{c} B\left(K_{i},R_{i},t_{i}\right)=𝟙\left(D_{i}\left(\Pi \left(K_{i}\left(R_{i}X+t_{i}\right)\right)\right)<\epsilon_{d}\right), \end{array}$$

where 𝟙 is the indicator function and $\Pi \left(\cdot \right)$ is the column-wise image projection operator, $\left[\lambda u,\lambda v,\lambda \right]\in R^{3}\to \left[u,v\right]\in R^{2}$. The view threshold $\epsilon_{v}$ and the distance threshold $\epsilon_{d}$ are set to $\epsilon_{v}=3$ and $\epsilon_{d}=3$, respectively, in our experiments, where the unit of $\epsilon_{d}$ is mm. The depth value was sampled by using bilinear interpolation.

**Figure 7:**
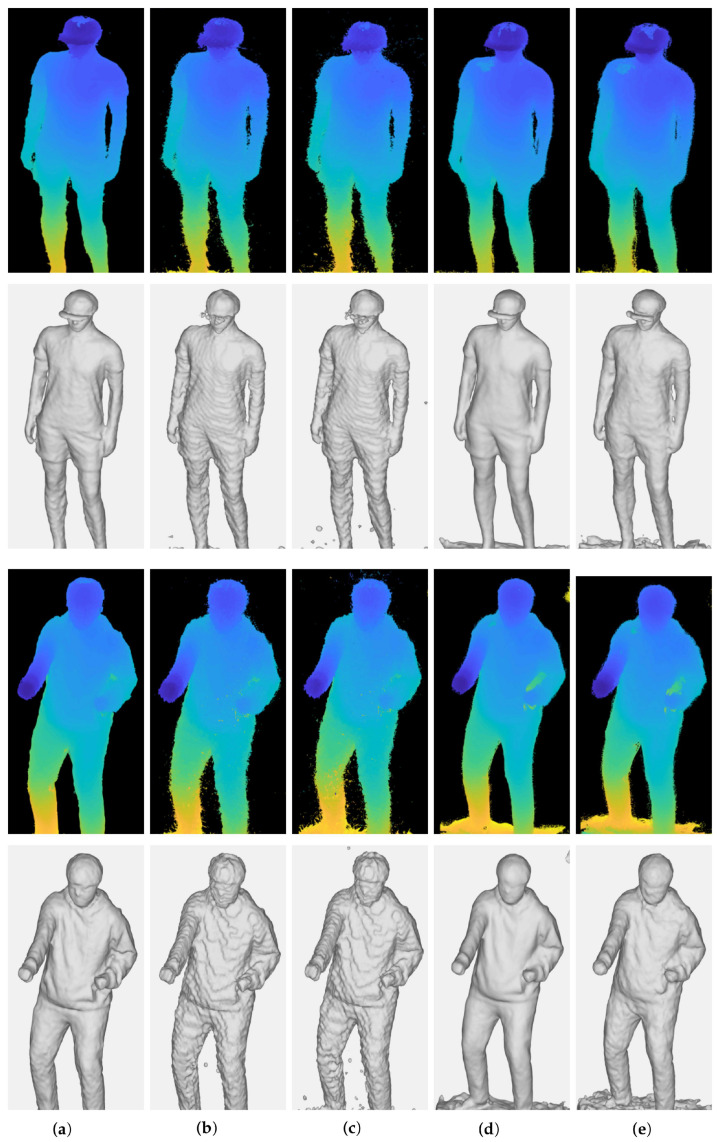
Qualitative results of 3D reconstruction after the outlier removal procedure according to the stereo matching algorithm. The noisy point cloud is removed from all sets of data and the noise surface of the 3D mesh is removed. (**a**) Census; (**b**) NCC; (**c**) SSD; (**d**) AANet; (**e**) Deep Pruner.

Figure 7 and Table 4 show quantitative and qualitative results after removing invalid 3D points in Figure 6 by the outlier removal scheme, respectively. The results show that inaccurate points and artifacts are significantly decreased without resolution loss by the outlier removal. This procedure is essential to accomplish an accurate reconstruction. In particular, it efficiently removes noisy background points from 3D points obtained from the deep learning-based methods.

Census and AANet stereo matching methods measure the least and comparable reprojection errors after outlier removal, as seen in Table 4. The reconstructed surfaces from the AANet method in Figure 7 are smoother than those from the Census, making less noisy reconstructed results. However, the smoothed surface also decreases the high-frequency details of target objects, such as wrinkles. Therefore, no superiority can be determined here as there is a trade-off between noise suppression and detail preservation.

### 4.6. Color Mapping

**Figure 8:**
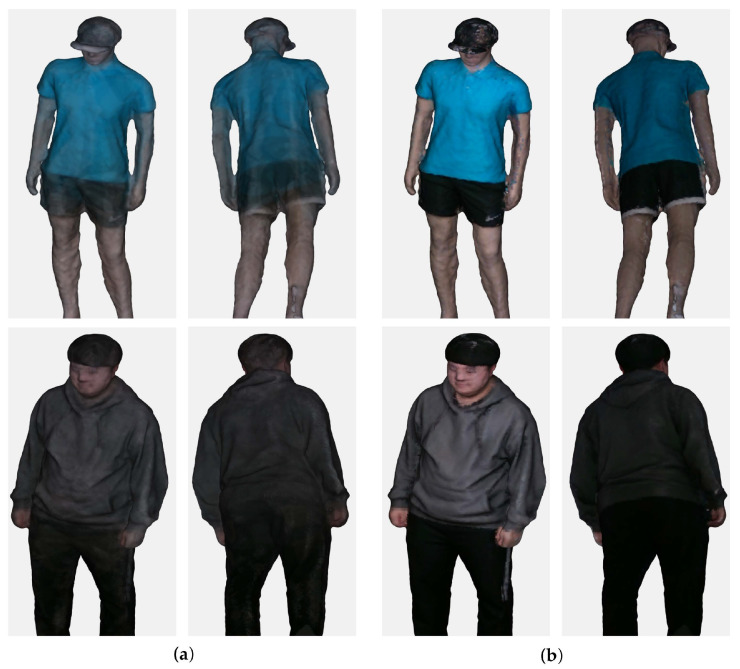
Colored 3D meshes. The 3D meshes are reconstructed using depth maps estimated by the Census stereo matching method. The median color mapping shows more reliable and accurate results than average colors. (**a**) Mean; (**b**) Median.

The vertex colors of the reconstructed mesh were mapped by reprojecting the vertices into images. Since the facial mesh composed of triangles was constructed in Section 4.4, vertex visibilities to each image could be determined using the z-buffering test [39] by projecting triangles. However, because the reconstructed mesh is an approximation from the integrated points X, many reprojected vertices into images do not tend to be mismatched. Assuming that the reconstructed mesh has *L* vertices, let $V\in R^{3\times L}$ be vertices of the reconstructed mesh and $v_{i}\in R^{1\times L}\left(1<i<N\right)$ be the visibility of the vertices to the ith camera obtained by the z-buffering test. The vertex colors $C\in R^{3\times L}$ obtained by reprojecting every image can be averaged as:

(10) $$\begin{array}{c} C=\frac{1}{\sum_{i}v_{i}}\sum_{i=1}^{N}v_{i}I_{i}\left(\Pi \left(K_{i}\left(R_{i}V+t_{i}\right)\right)\right) \end{array}$$

where $I_{i}$ is the color image captured from the ith RGB sensor. The product and division of $v_{i}$ are performed column-wisely along the row dimension. For simplicity of notation, this paper assumed that the camera’s intrinsic and extrinsic parameters of the color sensors were the same as the depth sensors in Section 4.4. The averaging scheme caused uneven colors due to mismatched vertices, as depicted in Figure 8a.

This problem is addressed by using a median color among those obtained from RGB images. The median colors provide significantly more accurate and reliable color mapping compared to the average colors by efficiently excluding outliers as depicted in Figure 8b.

### 4.7. Discussion

Through the experiments, we evaluated the effect of each sub-procedure on the 3D reconstruction results. Here, based on the experiments, we analyze and summarize the influences of the technical variable factors in each sub-procedure.

*RGB-D camera calibration* is an essential procedure used to integrate 3D points captured in the local sensors into the global coordinates. Thus, the accuracy of the camera calibration is directly related to the accuracy of the reconstructed object, which is made of the integrated 3D points. The checkerboard-based calibration outperformed spherical marker-based methods in the multi-sensor environment thanks to the strong priors of the checkerboard.

From the analysis of the *projector intensity*, it was found that the quality of depth estimation is dependent on the projector pattern dots in active stereo systems rather than the object’s texture. Moreover, the results provided an empirical finding—that the pattern projector intensity does not sensitively affect the quality of the depth.

The *Stereo matching algorithm* can directly affect the accuracy of depth estimation. The benchmark showed that Census and AANet methods predicted depths more accurately than the other stereo matching algorithms we evaluated. The Census method is a patch-based algorithm that estimates depths robustly on active images with high brightness variations using a non-parametric transformation. The AANet is a deep learning-based method that estimates depths using deformable convolution layers, flexibly coping with active image pattern dots.

In the *3D reconstruction* procedure, 3D points in the local sensor coordinates were transformed into the global coordinates; point sets from all sensors were integrated. The 3D object surface was reconstructed using the integrated point. However, significant artifacts can arise in the reconstructed surface due to noisy depths. Many noisy points existed in the background, especially when the depths were estimated using deep learning-based methods.

In the *outlier removal* procedure, these artifacts were efficiently manipulated by view-consistency. By reprojecting the integrated 3D points into multiple sensors, invalid points were efficiently detected and removed. The procedure can reconstruct a more accurate 3D surface.

The *color mapping* procedure maps colors to vertices of the reconstructed mesh. A vertex can be mapped to several RGB images in the multiple-camera environment. The mean of possible colors yielded significantly uneven and inaccurate results. This paper shows that clear and accurate colors could be obtained by using the median of the colors.

## 5. Conclusions

In this paper, we introduced a 3D reconstruction pipeline to reconstruct 3D objects from multiple active stereo sensors and presented examinations to improve the accuracy of the 3D reconstruction through analyses and benchmarks. These examinations provide helpful guidelines for high-quality 3D surfaces in the overall pipeline for 3D reconstruction using active stereo sensors. We believe our analyses, benchmarks, and guidelines will help people build their own studios and further the research related to 3D reconstruction [1,40].

Additionally, we discovered that external factors (e.g., projector patterns) affected depth accuracy. We provided essential considerations for 3D reconstruction using active stereo sensors and demonstrated that several factors in the reconstruction pipelines could significantly affect the quality of 3D reconstructed shapes. Many more considerations could increase the 3D quality. Moreover, we assumed off-the-shelf-sensors where pattern projectors and image resolutions were fixed. If we can handle them (e.g., pattern shape, pattern power, image resolution, gain, etc.), they will be considerations with great potential. We hope to research these topics in the future.

## Figures and Tables

**Table 1:** Reprojection errors of calibration methods (px).

Method	Mean	±Std.
Checkerboard [26]	0.5132	0.1213
Svoboda et al. [27]	0.8413	0.2231
Mitchelson et al. [28]	0.7482	0.2484

**Table 2:** Reprojection errors according to projector intensity (cm).

Intensity	30	60	90	120	150	180
Mean	2.4596	2.4020	2.4163	2.3736	2.3766	2.3690
±Std.	1.6251	1.5846	1.6123	1.5588	1.5700	1.5510
Intensity	210	240	270	300	330	360
Mean	2.3503	2.3756	2.3526	2.3326	2.3466	2.3446
±Std.	1.5293	1.5325	1.5498	1.5175	1.5356	1.5465

**Table 3:** Reprojection errors using stereo matching algorithms (cm).

Method	Census [30]	NCC [32]	SSD [33]	AANet [34]	DeepPruner [35]
Mean	2.3942	3.5395	3.5721	5.2124	5.9137
±Std.	1.7967	4.6474	5.0575	13.0765	11.8105

**Table 4:** Reprojection error after outlier removal (cm).

Method	Census [30]	NCC [32]	SSD [33]	AANet [34]	DeepPruner [35]
Mean	2.3565	3.1821	3.3148	2.3976	3.1293
±Std.	1.7051	3.3635	5.1835	1.8225	2.7937

## Data Availability

Not applicable.

## Abbreviations

The following abbreviations are used in this manuscript:

RGB-D	red, green, blue-depth
2D	two-dimensional
3D	three-dimensional
4D	four-dimensional
ToF	time-of-flight
IR	infrared
HD	high definition
CPU	central processing unit
GPU	graphics processing unit
OpenCV	open source computer vision
RMSE	root mean square error
NCC	normalized cross correlation
SSD	sum of squared differences
AANet	adaptive aggregation networks
